# Cloud-Based User Behavior Emulation Approach for Space-Ground Integrated Networks

**DOI:** 10.3390/s22010044

**Published:** 2021-12-22

**Authors:** Leiting Tao, Xiaofeng Wang, Yuan Liu, Jie Wu

**Affiliations:** School of Artificial Intelligence and Computer Science, Jiangnan University, Wuxi 214122, China; 6191910032@stu.jiangnan.edu.cn (L.T.); lyuan1800@jiangnan.edu.cn (Y.L.); jiewu20@jiangnan.edu.cn (J.W.)

**Keywords:** security of CPS, space-ground integrated network, cloud platform, user behavior emulation, model driven, epoll model, large-scale

## Abstract

Cyber-physical systems (CPSs) based on space-ground integrated networks (SGINs) enable CPSs to break through geographical restrictions in space. Therefore, providing a test platform is necessary for new technical verification and network security strategy evaluations of SGINs. User behavior emulation technology can effectively support the construction of a test platform. Given the inherent dynamic changes, diverse behaviors, and large-scale characteristics of SGIN users, we propose user behavior emulation technology based on a cloud platform. First, the dynamic emulation architecture for user behavior for SGINs is designed. Then, normal user behavior emulation strategy driven by the group user behavior model in real time is proposed, which can improve the fidelity of emulation. Moreover, rogue user behavior emulation technology is adopted, based on traffic replay, to perform the security evaluation. Specifically, virtual Internet Protocol (IP) technology and the epoll model are effectively integrated in this investigation to resolve the contradiction between large-scale emulation and computational overhead. The experimental results demonstrate that the strategy meets the requirement of a diverse and high-fidelity dynamic user behavior emulation and reaches the emulation scale of 100,000-level concurrent communication for normal users and 100,000-level concurrent attacks for rogue users.

## 1. Introduction

In recent years, cyber-physical systems (CPSs) have become a research hotspot since they make the entire system more reliable, efficient, and coordinated [1]. A CPS integrates computing, communication, and controlling systems, thus generating new ideas for studies of space-ground integrated networks [2]. However, many security problems emerge when a CPS is integrated with the space-ground integrated network (SGIN). On the one hand, security risks arise from the CPS itself. For instance, CPSs face many advanced persistent threat (APT) attacks [3], and massive user data information leads to a privacy security problem [4]. On the other hand, security risks exist in SGINs. For example, the complexity of the network structure makes it difficult to determine the source of the attack [5], and the SGIN is vulnerable to rogue program attacks during the operation of accessing and handover [6].

To resolve these security issues, many strategies have been implemented in recent years. For example, Sun et al. [3] have proposed a secure fuzzy testing approach for honeypot identification inspired by vulnerability mining to capture and predict network attacks against CPSs. In Ref. [4], the authors have effectively monitored resource access and prevented unauthorized information flow through an access control mechanism. In Ref. [7], the authors have proposed an event-based mobile vehicle cyber-physical security governance framework to achieve the goals of real-time event prediction. In Ref. [8], the authors have applied a cybersecurity knowledge graph to build an attack analysis framework for cyberattack-and-defense test platforms. In Ref. [5], the authors have constructed a cybersecurity knowledge graph for SGINs to attribute network attacks simply, effectively, and automatically. In terms of these new security strategies, providing an emulation platform is necessary for the performance testing of SGIN.

The literature related to this article can mainly be classified into three categories: the mathematical model of user behavior, the emulation of user behavior based on discrete event simulation, and the emulation of user behavior based on virtualization.

Regarding the mathematical model of user behavior, the authors in Ref. [9] have proposed a user behavior analysis method based on conversation emulation. This method used the ant colony optimization algorithm to model user behavior. However, the theoretical analysis method requires a large amount of calculation with a slow training process, which is not suitable for large-scale user behavior emulation experiments. In Ref. [10], the authors have proposed a web behavior classification model for the collective user. The user identification method based on the computer mouse dynamic behaviors has been investigated in Ref. [11]. A top-down layered method has been proposed in Ref. [12] to build a behavior traffic model in the satellite terminal. In Ref. [13], the authors have proposed a multiple-strategy differential privacy framework on STF (MDPSTF) for user traffic model analysis. In Ref. [14], the authors have proposed a GPU-accelerated parallel H-ELM to efficiently process large-scale data. It can provide support for user behavior models. The above efforts have provided a theoretical analysis basis for the user behavior emulation. However, such research cannot establish a systematic emulation of user behavior.

Regarding the emulation of user behavior based on discrete event simulation, in Ref. [15], the authors have constructed the network emulation model of the campus network and analyzed the user behavior of the networks by using the OPNET simulation platform. In Ref. [16], the authors have designed an emulation system in the wireless heterogeneous network (HetNet) based on network simulator 3 (NS3) and verified the behavior of wireless access users. Nicol [17] has proposed a hybrid discrete-continuous model that minimizes network worm’s execution time to achieve large-scale emulation of network worm behavior. The authors in Ref. [18] have proposed a multilayer network traffic model based on time-space correlation and Markov transfer to construct user groups related to traffic time and space based on OPNET. However, user behavior emulation based on discrete event simulation cannot load real business systems and traffic. Moreover, it faces certain limitations in emulation fidelity.

Regarding the emulation of user behavior based on virtualization, in Ref. [19], the authors have constructed an emulation platform for the performance evaluation of crowdsensing applications based on virtualization. In Ref. [20], the authors have proposed an estimation mechanism that is based on analyzing the interaction between user behavior and network performance and verified the effectiveness through emulation experiments based on virtualization. A network traffic generator based on container virtualization technology has been proposed in Ref. [21] to emulate users’ web crawling behavior. In Ref. [22], the authors have studied the utilization of paravirtualization techniques merged with systems management tools to build an automated emulation framework for grid experiments and validated the effectiveness and advantages through user behavior experiments. In Ref. [23], the authors have proposed a wireless data service providing framework in C-RAN aiming to provide user service in C-RAN in a more efficient way based on mobile cloud computing (MCC). The investigations mentioned above have improved the simulation fidelity. However, it is mainly oriented to the traditional Internet, and there is a lack of research on SGIN users.

User behavior emulation based on virtualization technology has been turning to the mainstream research direction. The OpenStack cloud platform, which integrates full virtualization technology (e.g., kernel virtual machine, KVM) and lightweight virtualization technology (e.g., Docker), has become a better choice for realizing user behavior emulation due to its low cost, security, flexibility, credibility, and other advantages.

Providing a realistic emulation environment of user behavior is necessary for the security technology test of the SGIN emulation platform. However, there are still problems with user behavior emulation, e.g., single-user behavior, small scale, and poor fidelity.

In this article, we propose a large-scale user behavior emulation technology in SGINs based on the OpenStack cloud platform as a multiscale virtualization technology. To meet the needs of dynamic user behavior emulation, we integrate a systems tool kit (STK) [24] with OpenStack. By utilizing STK satellite orbit model data to online drive the emulation network link state, we can dynamically control the network environment in which the user behavior emulation is located. Considering the characteristics of multiple user types and diverse behaviors, we study the emulation technology of normal user behavior based on the real-time drive of the user behavior model. Additionally, the emulation technology of rogue user behavior based on traffic replay is analyzed. To realize large-scale user behavior emulation, virtual Internet Protocol (IP) technology is adopted to achieve a single network card and multiple users. Furthermore, the epoll model is employed to improve efficiency in large-scale communication.

Overall, the main contributions of this article are summarized as follows:

- Through the seamless integration of STK and OpenStack, the dynamic emulation needs of user behavior can be met;
- By studying the emulation technology of normal user behavior, based on the real-time drive of the user behavior model, and rogue user behavior, based on the traffic replay, the realistic emulation of diversified user behavior is realized. A delay compensation strategy is proposed to improve replay fidelity;
- Virtual IP technology and the epoll model are integrated effectively, which solves the problem of high resource consumption and low communication efficiency in large-scale user behavior emulation.

The rest of this article is organized as follows: Section 2 introduces the emulation system of the SGIN. In Section 3, an implementation of user behavior emulation in SGINs is proposed and analyzed. Experimental verification is carried out in Section 4. We conclude the entire article in Section 5.

## 2. User Behavior Emulation System for the SGIN 

Interconnected and multiple heterogeneous networks form the SGIN. Its network is large in scale, complex in structure, and highly dynamic [25]. Its users include the terrestrial Internet, a mobile communication network. Access users are large in scale, diverse in behavior, and large in concurrent business [12]. Given the characteristics of SGIN users, based on the cloud platform and limited fixed computing resources, realizing the highly dynamic, diverse behavior and large-scale emulation of SGIN users remains a problem to be solved. Therefore, this paper constructs a user behavior emulation system for the SGINs to meet the above emulation requirements.

**Figure 1:**
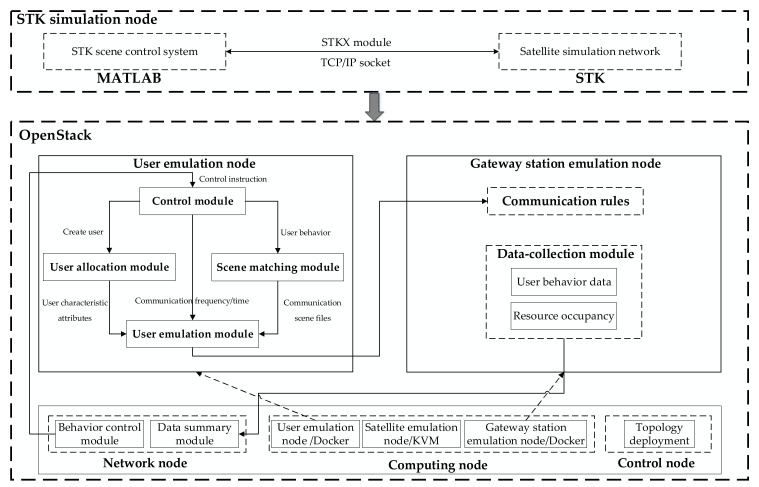
Emulation architecture of the SGIN user behavior.

Figure 1 shows the overall architecture of the SGIN user behavior emulation system, including the STK simulation node and the OpenStack cloud platform. The STK simulation node is responsible for the data level of the entire emulation architecture and provides the source data for satellite network emulation on the OpenStack cloud platform. The OpenStack cloud platform is responsible for the control level of the entire emulation platform. After receiving the emulation data, it controls the space-ground integrated emulation network topology and satellite emulation link characteristics in real time. See Section 3.1 for the seamless integration of STK and OpenStack.

The OpenStack cloud platform includes a control node, a network node, and several computing nodes. The control node is responsible for the planning and deployment of the network topology. The network node deploys a data summary module for real-time statistical information and coordinates user emulation nodes through the behavior control module. Computing nodes use multiscale virtualization technology to create satellite emulation nodes, gateway station emulation nodes, and user emulation nodes. Among these nodes, the satellite emulation node is constructed with KVM to ensure the stability of the satellite link; the gateway station emulation node and user emulation node use Docker to reduce resource utilization. The gateway station emulation node deploys communication rules and data-collection modules. User communication traffic is forwarded through the communication rules. User behavior emulation data and resource loss information are obtained through the data-collection module and pushed to the data-summary module to realize information acquisition and analysis. The user emulation node integrates the control module, the user allocation module, the scene matching module, and the user emulation module. This node receives user behavior emulation instructions to achieve the corresponding emulation requirements. The functions of each module and instructions for how to communicate between modules are as follows:

- **Control module:** The control module controls the entire procedure, including the user allocation module, the scene matching module, and the user emulation module. It is the control center for the realization of the entire user behavior emulation. After the module receives the instructions issued by the behavior control module in the network node, it controls other modules to coordinate work according to the instruction information to complete the user behavior emulation;
- **User allocation module:** The user allocation module is the main body of user behavior emulation, and users adopt user identifications (IDs) as the distinguishing standard, which is the primary key in the user pool. To ensure the fidelity of users, the user attributes also include the user IP, user name, user password, etc. After the module receives the user creation instruction issued by the control module, it parses the instruction to obtain the required user information and then obtains the user’s characteristic attributes from the corresponding user pool and transfers them to the user emulation module;
- **Scene matching module:** The scene matching module controls the communication mode and communication process of the emulated user. After the module receives the user behavior instruction issued by the control module, it obtains the specified user behavior model from the instruction. It selects the matching user behavior scene file and transfers it to the user emulation module;
- **User emulation module:** The user emulation module is an emulation module of user behavior communication. This module personalizes the generation of user behavior by receiving the user characteristic attributes, communication scene files, frequency, time, and other transmitted information. Among this information, the normal user behavior generates protocol traffic based on the communication protocol. The Internet user uses a hypertext transfer protocol (HTTP) and a file transfer protocol (FTP) to realize web browsing and file transfer behaviors. The mobile terminal user uses SIPp, a configurable traffic generation tool that meets Session Initialization Protocol (SIP) standards, to emulate calls and short message services (SMSs). Moreover, normal user business traffic is generated according to the rules of the user behavior model. The normal user behavior model-driven strategy is presented in Section 3.2; rogue users generate rogue attack traffic based on traffic replay, and the specific implementation is presented in Section 3.3. Additionally, large-scale emulation experiments can be carried out according to the requirements, and the specific implementation is presented in Section 3.4.

## 3. Implementation for User Behavior Emulation of the SGIN

### 3.1. Seamless Integration of STK and OpenStack

**Figure 2:**
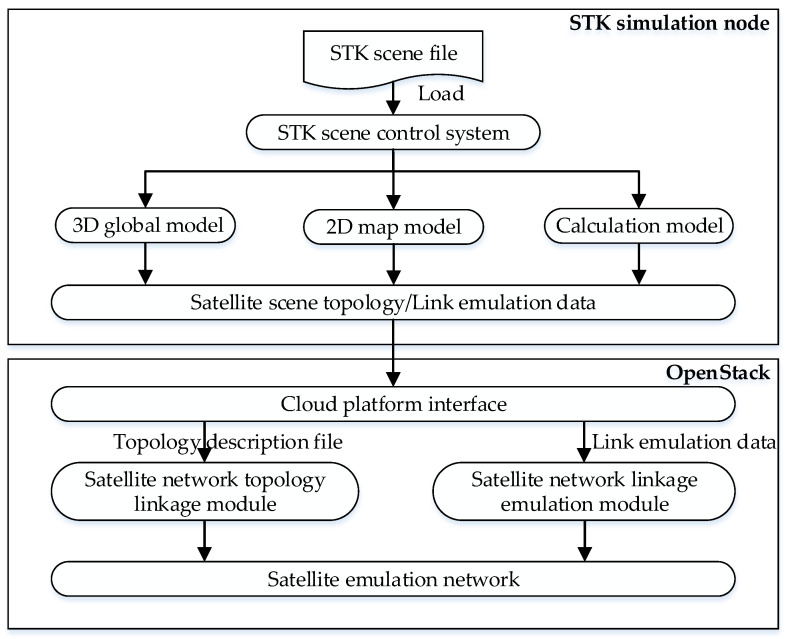
STK dynamic control satellite emulation network flow chart.

STK’s satellite network calculation model can effectively describe dynamic characteristics such as network topology and link characteristics [26,27]; it can accurately analyze the performance and behavior of complex networks [28]. This network offers the advantages of simple management and high scalability. However, it cannot emulate the real protocol and business traffic. Network emulation based on the OpenStack cloud platform can run real business traffic, and the emulation results have high fidelity and scalability. However, the OpenStack cloud platform lacks the support of the related satellite network calculation model. In this regard, this paper studies the seamless integration of STK and OpenStack to realize the dynamic emulation of SGINs to meet the dynamic emulation needs of user behavior. The specific procedure is shown in Figure 2.

The STK scene control system loads the STK scene file. It sets the relevant parameters of the emulation scene in the STK, mainly including the STK scene ephemeris time, rain attenuation model, atmospheric absorption model, scene operation step length, and other parameters. Moreover, it reads the satellite network topology of the 3-dimensional (3D) global model and 2-dimensional (2D) map model in STK to generate the satellite scene topology.

Using the calculation model in the STK obtains link emulation data, and the on-off of the satellite link is calculated based on visibility modeling between objects. The satellite link delay can be calculated with Equation (1), where D is the link length, f(t) is a time-varying function, T is the link length change period, $t_{d}$ is the link delay, and c is the propagation speed of electromagnetic waves in the vacuum.

(1) $$\left\{\begin{array}{l} D=f(t),t\in T\\ t_{d}=\frac{D}{c} \end{array}\right.$$

Given the signal energy per bit $E_{b}$ and the power spectral density of the noise $N_{0}$, the bit error rate (*BER*) of the link can be expressed as

(2) $$BER=\frac{1}{2}erfc(\sqrt{\frac{E_{b}}{N_{0}}})$$

where the complementary error function erfc(.) is defined as

(3) $$erfc(x)=\frac{2}{\sqrt{\pi }}\int_{x}^{\infty }e^{-n^{2}}dn$$

The source data of satellite link emulation in real time are pushed to computing nodes on the cloud platform at a specific time step through the cloud platform interface. The satellite network topology linkage module is responsible for parsing the satellite scene topology, including node information and network segment information. The node information mainly includes the node type, node size, and satellite mirroring type. The network segment information includes the network name and network subnet. The satellite network linkage emulation module is responsible for dynamic and real-time control of the satellite link characteristics in the cloud platform based on the satellite link emulation data, mainly including the link on-off, link delay, and link bit error rate.

### 3.2. Normal User Behavior Model-Driven Strategy

The user behavior model-driven strategy needs to be designed based on the theoretical model of the algorithm by extracting user information data and behavior rules, forming a description script of user behavior emulation, and driving the user behavior emulation in real time. In terms of the user behavior model, user behavior is random, and the relationship between users is independent and irrelevant. Dombry et al. [29] indicated that user behavior data traffic conforms to the Poisson distribution model, therefore this paper proposes a Poisson distribution algorithm based on random users to drive user behavior in real time. The specific implementation can be seen in Algorithm 1.
**Algorithm 1:** Poisson Distribution Algorithm Based on Random Users.**Input:** Poisson distribution expectation: *λ*; Number of users: *m*; User behavior emulation times: *n*.
**Output:** Array of the user ID: *p[1…n][1…n]* **begin** 1.      rand_max is initialized to the maximum number of users 2.      **for** i ←0 to n by 1
**do** 3.      $u\leftarrow Rand\left(0,1\right)\;/rand\_max$
*// generate a uniform distribution of [0, rand_max]* 4.      x←0 5.      $cdf\leftarrow exp\left(-1\;*\;\lambda \right)$ 6.      while u>=cdf and  x<=m
*// get the number of users in this round* 7.       x←x+1 8.       pdf←1 9.       for j←0 to x by 1 do   *// update the probability* 10.         pdf←pdf * λ / j 11.       **end for** 12.       $cdf\leftarrow cdf+pdf\;*\;exp\left(-1\;*\;\lambda \right)$ 13.        **for** k ←0 to x by 1 **do**     *// get random user IDs* 14.                  **while true** 15.         $user_{ID}\leftarrow mod\left(Rand\left(0,1\right),m\right)+1$ 16.         $\mathrm{if}\;user\_ID\;\mathrm{is}\;\mathrm{not}\;\mathrm{in}\;p\left[i\right]$ 17.               **break** 18.              $p\left[i\right]\left[j\right]\leftarrow user\_ID$ 19.          **end for** 20.   **end for** **end**

### 3.3. Rogue User Behavior Emulation Based on Traffic Replay

**Figure 3:**
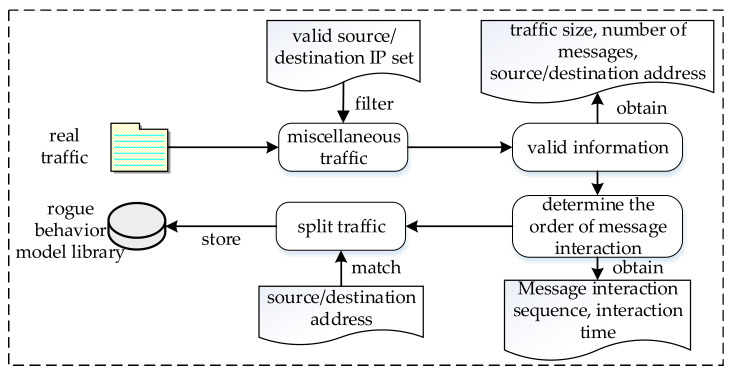
Rogue traffic preprocessing flow chart. The real traffic is filtered, split, and other operations; the rogue traffic and its characteristic information are stored in the rogue behavior model library.

The implementation of rogue user behavior emulation technology mainly includes rogue traffic preprocessing and rogue attack emulation. Figure 3 shows the rogue traffic preprocessing process.

Once real rogue traffic is successfully obtained and preprocessed, the preconditions for rogue attack emulation have been completed. The specific rogue attack emulation flow is as follows:

1. The port is opened to monitor the rogue user emulation node. After receiving the rogue attack command, the attack parameters, including the rogue attack type, source/destination IP address, number of attacks, attack rate, and other information, are parsed;
2. According to the attack type, the qualified traffic and its related characteristic information from the rogue behavior model library are extracted. Moreover, the traffic message’s source/destination IP address and the destination Media Access Control (MAC) address are modified to the MAC address of the network card connected to the virtual router;
3. For two-way traffic, the interaction order and interaction time of the traffic need to be sent to the corresponding two rogue user emulation nodes;
4. After handling rogue traffic, we can set the number of rogue attacks and the attack rate and then replay the traffic in the rogue user behavior network.

For two-way traffic, the most critical goal is to ensure that the timing state of the data packets and the content of the message are consistent with the original traffic. This goal is also one of the most important indicators for evaluating the fidelity of the emulation. However, during the replay process, the replay effect may be affected by link delay, jitter, and replay node processing delay, causing out-of-sequence data packets. Therefore, this paper proposes a delay compensation strategy under the premise of ensuring the accuracy of the message sequence as far as possible to minimize the replay time error. The specific strategy is as follows:

Before the emulation experiment, equal numbers of traffic files with fixed intervals are set for replay. The time intervals Δo and Δr of each pair of adjacent packets in the original traffic and the replay traffic are compared to obtain an error average. The calculation equation is as follows:

(4) $$Error_{\mathrm{avg}}=\frac{\sum_{i=1}^{n}\left(|\Delta ri-\Delta oi|\right)}{n}$$

The average error obtained is used to compensate for the original traffic. The specific operation is as follows: the sequence is traversed from the second packet in the original traffic. When the message satisfies $\Delta oi<Error_{\mathrm{avg}}$ and has a different side from the previous message, the current message stamp Tsi is compensated according to the following equation. The average error obtained is used to compensate for the original traffic.

(5) $$Tsi=Tsi-1+Error_{\mathrm{avg}}$$

Moreover, the timestamp of the packet is updated until the last packet. It is ensured that each packet interval is not less than the average error, thus reducing the error caused by the delay in replay delay.

### 3.4. Large-Scale User Behavior Emulation

**Figure 4:**
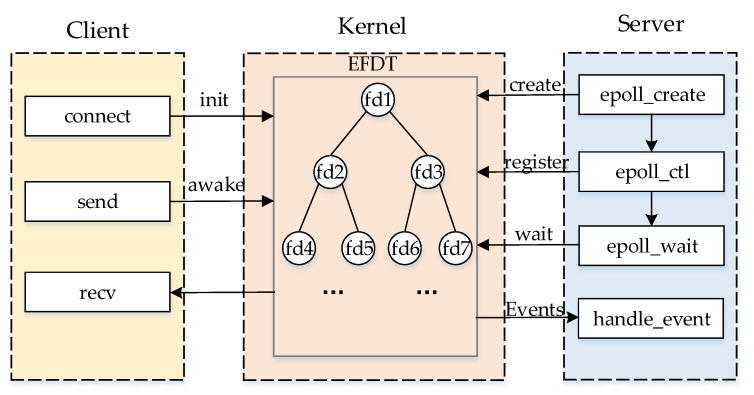
Epoll model communication flow chart.

Large-scale user behavior emulation puts forward requirements for large-scale user and user high concurrent communication. To solve the above technical difficulties, this paper proposes the following scheme:

- **Large-scale user implementation:** To conserve limited physical resources and solve the problem that a cloud host has only one network card and can emulate only one user, this paper combines the technical idea of a single network card with multiple IPs [30]. Multiple virtual IPs are added to a cloud host to ensure that the emulated users have their independent IP addresses by expanding virtual information for the network card. With the help of a routing protocol, the destination network routing table is automatically added to the node to realize intercommunication between the virtual IP and the real IP and the virtual IP and the virtual IP on different hosts. Communication between users is achieved by establishing a transmission control protocol (TCP) connection, which is combined with the TCP connection mechanism by establishing a virtual user pool. When a user initiates a connection, the user is first retrieved from the pool, and the user information is encapsulated in a user request packet to achieve user communication;
- **User high concurrent communication implementation:** To meet the high concurrent communication needs of large-scale SGIN users, the server uses the epoll model to realize the input/output (I/O) multiplexing of socket stream read-write operations and handle the concurrent communication data of large-scale users. Figure 4 shows the communication procedure based on the epoll model. Compared with the traditional select/poll mechanism, the epoll model does not need to traverse the entire monitored descriptor set. When the File Descriptor (FD) event status registered in the Epoll File Descriptor Tree (EFDT) changes, the kernel directly returns these events in the Events array, thus the epoll monitoring efficiency does not decrease significantly as the number of monitored events increases. Moreover, there is no limit to the number of events monitored. This limit is related to only system resources. The epoll model can achieve user behavior drive more efficiently, solve the numerical limit of high concurrent communications for large-scale users, and improve emulation efficiency.

## 4. Experiment

This paper uses an OpenStack distributed architecture as an emulation platform to build a low-cost and large-scale emulation experimental environment. The version of OpenStack is Mitaka, and the experimental environment is set as in Table 1. The satellite emulation nodes realize the transparent forwarding function of the satellite. The gateway station emulation nodes integrate communication management services. The user emulation nodes integrate user behavior function services.

**Figure 5:**
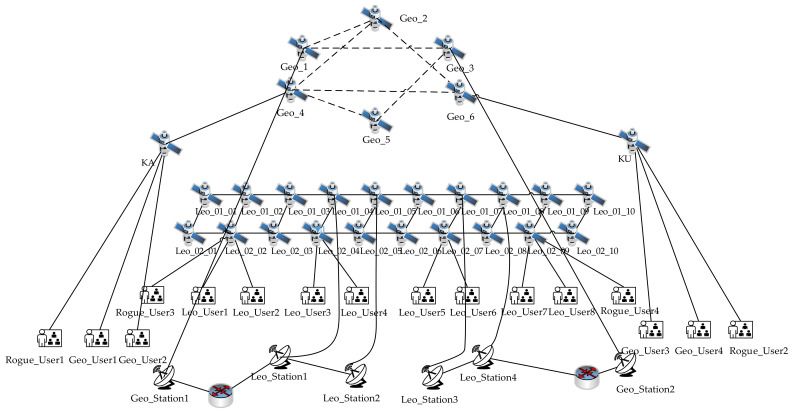
Experimental topology. It contains 20 low-orbit satellites, 6 high-orbit satellites, 2 communication satellites, 4 Internet user groups, 8 mobile terminal user groups, 4 rogue user groups, 2 Internet gateway stations, and 4 mobile terminal gateway stations.

An automated deployment scheme connects the emulation node to the emulation network to construct an experimental topology. The specific experimental topology is shown in Figure 5. Geo represents the high-orbit satellite; KA and KU stand for the communication satellite; Leo stands for the low-orbit satellite; Leo_Station represents the mobile terminal gateway station; Geo_Station stands for the Internet gateway station; Leo_User represents the normal mobile terminal user; Geo_User stands for the normal Internet user; and Rogue_User represents the rogue user. When automatically assigning resources, the satellite node allocates 1 virtual central processing unit (VCPU) and 1 GB of memory, the gateway station node allocates 4 VCPUs and 4 GB of memory, and the user emulation node allocates 2 VCPUs and 2 GB of memory.

### 4.1. Dynamic Emulation Experiment of the SGIN

In order to verify the effectiveness of the dynamic control technology proposed in this article, we select Geo_User1 and Geo_User3 from Figure 5 and then control Geo_User1 and Geo_User3 for web browsing and file transfer behavior. The user behavior traffic rate is collected on Geo_Station1 and Geo_Station2 every 1 s. At the same time, the positions of Geo_4 and Geo_5 are switched through the STK every 60 seconds.

**Figure 6:**
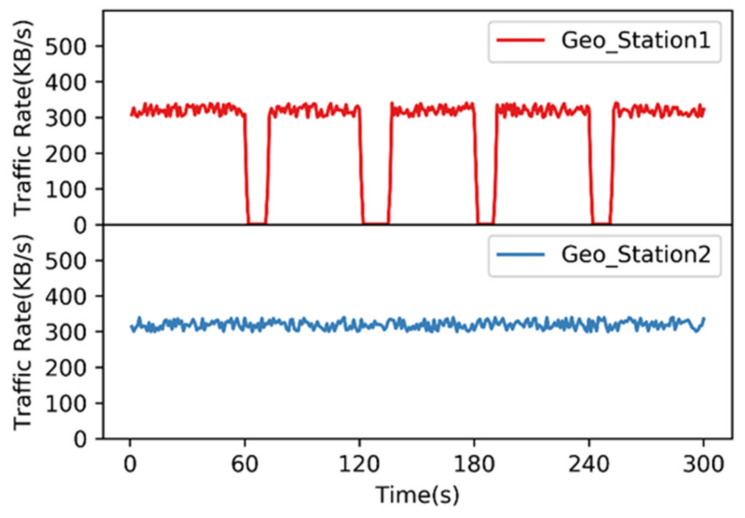
Dynamic emulation experiment of user behavior under STK control. The above experimental data measures the change in the rate of user request traffic received by Geo_Station1 as the server in the dynamic switching situation; the following experimental data measures the change in the rate of user request traffic received by Geo_Station2 as the server in the non-switching situation.

In Figure 6, when the satellite position is not switched, the traffic data received by the gateway station is relatively stable, which means that the user’s communication behavior is proceeding normally. When Geo_4 and Geo_5 are switched, the connected Geo_User1 communication service is affected and cannot be performed for a period of time. Geo_User3 does not communicate through the satellites mentioned above, thus communication services are performed normally. As the satellite position switch is completed, Geo_User1 establishes a connection with another satellite, and communication returns to normal. Based on the above experimental results, the technology adopted in this article can realize the dynamic control of the satellite emulation network and can more realistically reproduce user communication under the SGIN.

### 4.2. Normal User Behavior Model-Driven Experiment

This experiment is based on the user behavior model in Section 3.2, drives the emulation node, and realizes the function of scene reproduction. We select Geo_User3, Leo_User5 and Leo_User7 from Figure 5, and 200 users are created at each node as experimental subjects. Then, each user is controlled to generate user behavior traffic satisfying the Poisson distribution expectation of 100. Finally, the number of data packets arriving at Geo_Station2 and Leo_Station3 are collected every 1 s, and the corresponding statistical analyses are carried out according to different intervals. The whole model-driven emulation experiment lasts for 100 s.

**Figure 7:**
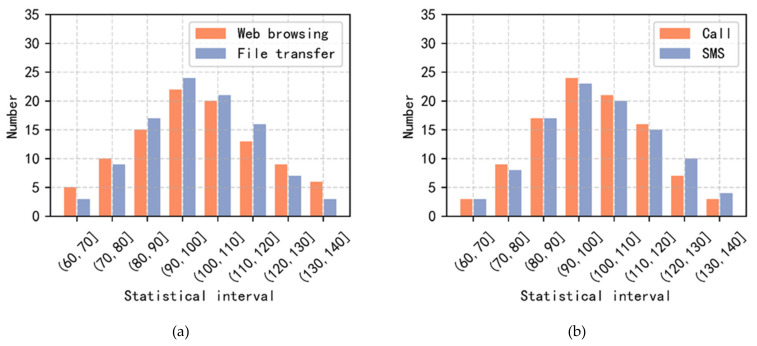
Statistical histogram of the number of access successes for normal user behaviors. (**a**) Internet user; (**b**) Mobile terminal user. The statistical histogram is obtained by counting the number of data packets reached per second and then dividing according to the number interval.

The experimental results are shown in Figure 7. Counting the number of data packets per second in the 90–110 interval as the most frequent, the user behavior model law conforms to the Poisson distribution law. The above experimental results show that the user behavior model-driven strategy studied in this paper can effectively drive emulation nodes and realistically reproduce user communication scenes.

### 4.3. Rogue User Behavior Emulation Experiment

**Figure 8:**
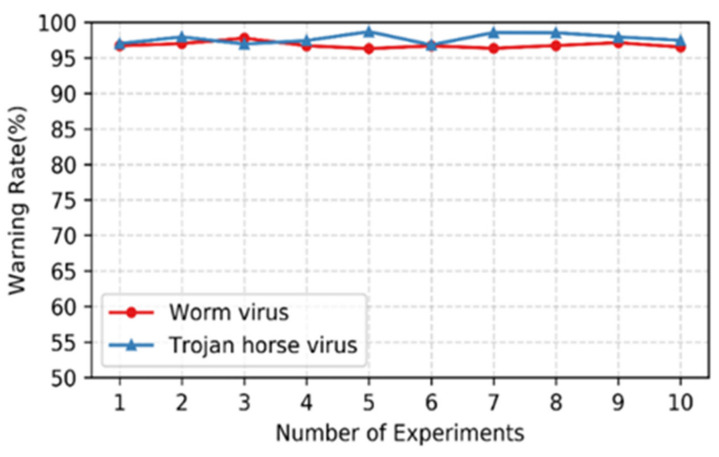
Emulation experiment of rogue virus attack based on traffic replay. The success rate of worm and Trojan virus attack emulation is calculated by the number of warnings generated by snort.

We select Abn_User1 and Geo_User1 from Figure 5. Furthermore, Rogue_User1 is controlled to carry out rogue attacks involving the Worm virus and the Trojan horse virus on Geo_User1. In addition, the snort is installed on satellite node KA in the topology to detect rogue traffic. For different types of rogue traffic, corresponding rule files are added to establish the characteristics of traffic packets. Finally, the number of rogue attacks in a single experiment is set as 10,000, and the number of experiments is ten times. Moreover, the warning rate is quantified. The experimental results are shown in Figure 8. The warning rate of rogue user behavior realized by the method in this paper is above 95%. The average warning rate of the Worm virus is 97.1% and that of the Trojan horse virus is 98.1%.

At the same time, in order to verify the effectiveness of the delay compensation strategy proposed in this paper, five sets of rogue traffic files are replayed between Rogue_User3 and Rogue_User4. The numbers of these data packets are 20,000, 50,000, 100,000, 200,000, and 300,000. The time error value obtained is compared with the time error value generated by the traffic replay software Tcpreplay [31].

**Figure 9:**
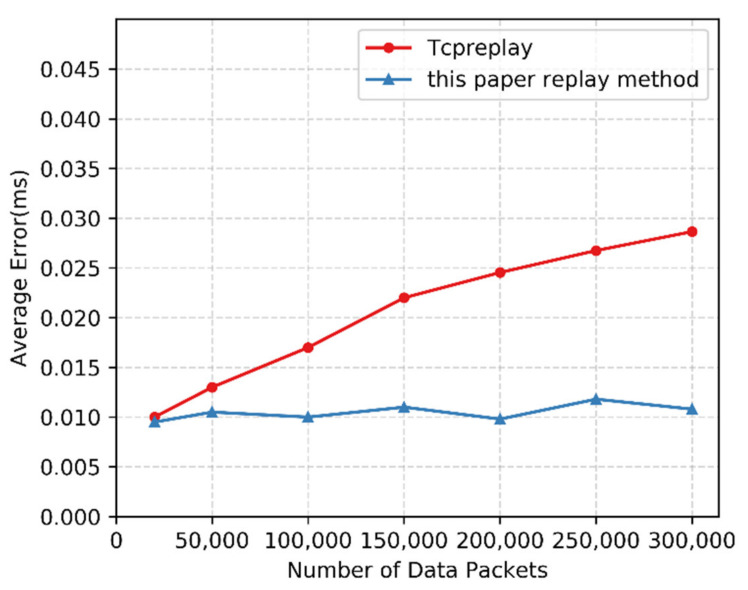
Comparison of the average error between the method in this paper and the Tcpreplay replay method.

The experimental results are shown in Figure 9. With the increase in data packets, the error of replay through Tcpreplay gradually increases. The error of the method used in this paper is maintained at a low level, which shows that the method of this paper has certain advantages in replay fidelity compared with the Tcpreplay replay method.

It can be observed from the experimental results that the method studied in this paper can achieve high-fidelity emulation of rogue user behavior. At the same time, it can provide environmental support for SGIN security evaluations.

### 4.4. Large-Scale Emulation Experiment of User Behavior

The communication system in the real environment needs to provide services for large-scale users and experimental support for constructing the SGIN, thus the emulation platform should have abilities suitable for large-scale experiments. In this paper, large-scale emulation experiments are carried out for normal user behavior. Based on the emulation topology built in Figure 5, large-scale emulation users are created and deployed on multiple normal user nodes, 1000 users are created every second, and such users are driven to generate various behavior traffic. The number of concurrent users in a period is counted by collecting communication traffic in the gateway station. Considering the pressure for the server by large-scale user concurrent communication, the memory consumption of the gateway is based on real-time statistics.

**Figure 10:**
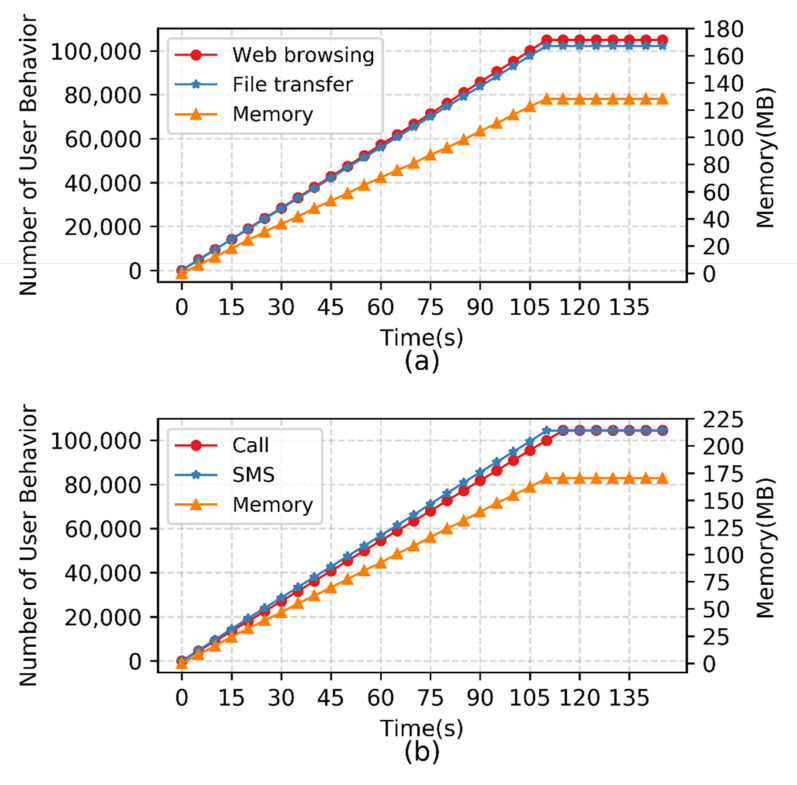
Large-scale emulation experiment on normal user behavior. (**a**) Internet user; (**b**) Mobile terminal user.

The experimental results are shown in Figure 10. As the number of concurrent users gradually increases, the communication demand pressure of the gateway station gradually increases, and the corresponding memory consumption increases accordingly. When the number of concurrent users reaches a stable value, the memory consumption of the gateway station tends to become stable.

**Figure 11:**
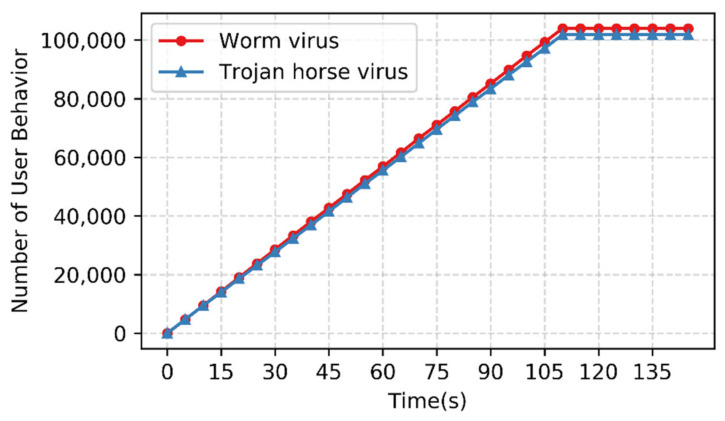
Large-scale emulation experiment on rogue user behavior.

At the same time, the communication network may encounter large-scale rogue attacks, therefore it is necessary to conduct large-scale rogue attack emulation experiments. The emulated users are deployed on rogue user nodes, 1000 users are created every second, and then the rogue users are driven to launch attacks on the normal user nodes. Finally, the concurrent number of rogue attacks within a period is collected from the attacked node. The experimental results are shown in Figure 11. With increasing time, the number of concurrent attacks by the Worm virus and the Trojan horse virus can reach 100,000.

The experimental results show that the method designed in this paper can meet the emulation scale of 100,000-level concurrent communication for normal users and 100,000-level concurrent attacks for rogue users.

### 4.5. Discussion

The results of the dynamic emulation experiment in Section 4.1 show that our emulation environment can achieve the real-time emulation of satellite networks, which can meet the requirements of user behavior emulation. The second experiment in Section 4.2 indicates that the proposed model-driven strategy conforms to the law of the Poisson distribution model, which shows that the strategy can effectively reproduce the user communication scene and improve the emulation fidelity. The third experiment in Section 4.3 on rogue user behavior emulation shows that the traffic replay technology proposed in this paper can realize the fidelity reproduction of rogue virus attacks to provide environmental support for security evaluation. The final experiment in Section 4.4 shows that the virtual IP technology can generate large-scale users, and the epoll model is applied to solve the problem of low communication efficiency in large-scale user behavior emulation.

## 5. Conclusions

With the background of SGINs and CPSs, this paper has proposed an emulation method for large-scale user behavior based on the cloud platform. It has built a user communication environment for SGINs. Dynamic control of the satellite network environment has been achieved through STK. The user behavior model has been adopted to drive business traffic generation, and the rogue attacks have been realistically reproduced through the traffic replay technology. 

However, due to the lack of investigation and research on the security issues of the emulation platform, the emulation platform proposed in this paper still faces many security threats. Given the problem, our future research will enhance the security of SGINs via other novel approaches based on the user communication environment and rogue attack environment constructed in our investigation.

## Figures and Tables

**Table 1:** OpenStack nodes configuration information.

Node	CPU	Memory
Control node	Intel(R)Xeon(R)CPU E5-2620 v4 × 2	64 GB
Network node	Intel(R)Xeon(R)CPU E5-2609 v3 × 2	16 GB
Computing node 1	Intel(R)Xeon(R)CPU E5-4607 v2 × 4	32 GB
Computing node 2	Intel(R)Xeon(R)CPU E5-2620 v2 × 2	32 GB
Computing node 3	Intel(R)Xeon(R)CPU E5-2620 v2 × 2	32 GB

## Data Availability

Not applicable.
